# Highly Elastic Melamine Graphene/MWNT Hybrid Sponge for Sensor Applications

**DOI:** 10.3390/molecules27113530

**Published:** 2022-05-31

**Authors:** Christos Fragkogiannis, Apostolos Koutsioukis, Vasilios Georgakilas

**Affiliations:** Department of Material Science, University of Patras, 26500 Patras, Greece; up1061363@upnet.gr (C.F.); up1057091@upatras.gr (A.K.)

**Keywords:** graphene, carbon nanotubes, melamine sponge, strain sensor, piezoresistivity

## Abstract

The rapidly increased interest in multifunctional nanoelectronic devices, such as wearable monitors, smart robots, and electronic skin, motivated many researchers toward the development of several kinds of sensors in recent years. Flexibility, stability, sensitivity, and low cost are the most important demands for exploiting stretchable or compressible strain sensors. This article describes the formation and characteristics of a flexible, low-cost strain sensor by combining a commercial melamine sponge and a graphene/carbon nanotubes hybrid. The composite that emerged by doping the highly elastic melamine sponge with a highly conductive graphene/carbon nanotubes hybrid showed excellent piezoresistive behavior, with low resistivity of 22 kΩ m. Its function as a piezoresistive material exhibited a high sensitivity of 0.050 kPa^−1^ that combined with a wide detection area ranging between 0 to 50 kPa.

## 1. Introduction

Graphene is a unique 2D carbon nanomaterial with exceptional properties such as mechanical strength and flexibility, electrical and thermal conductivity, high specific area, and chemical stability [1,2]. It is very stable and inert under normal or mild conditions such as temperature, oxygen, humidity, mild acidic, or alkaline environment. Graphene can be functionalized by a plethora of organic reactions affording interesting derivatives [3]. Therefore, graphene and its derivatives have been investigated in a great variety of applications, including sensors [4,5,6], conductive inks [7,8], bioapplications [9], batteries, and supercapacitors [10,11,12], etc. In most of these applications, the 2D structured graphene nanosheets are necessary to be combined with bulk materials such as polymers, ceramics, or metals. Consequently, they form, for example, by mixing applicable composites or by deposition or printing modified surfaces of layered 3D devices [13,14,15,16].

One of the most interesting 3D structures that exploit the properties of graphene is the aerogel. An ideal graphene structured aerogel could combine the unique electrical and thermal conductivity and the elasticity of graphene with the large surface area and the porosity offered by the aerogel structure [17,18,19,20]. Graphene oxide (GO) is a hydrophilic derivative of graphene derived from graphite’s oxidative treatment. It has received tremendous attention since it is easily prepared in large quantities in monolayers, and although it is a nonconductive material, it becomes conductive by several simple reduction processes [21]. GO has been shown in the literature that it can be organized into lightweight, porous, 3D structures such as aerogels, foams, or sponges in several ways. However, some structural disadvantages, mainly fragility and mechanical instability, restrict their use in several applications such as supercapacitors, batteries, membranes for water purification or gas separation, and electromagnetic interference shielding [22,23,24].

To overcome these disadvantages and prepare a stable, compact graphene aerogel, the combination of graphene with other materials has been examined. Recently, our group showed the successful reinforcement of graphene aerogel by carbon fibers [25,26]. A different approach has been followed in several works, where melamine foam or sponges were used as a stable and highly flexible skeleton to develop graphene-based porous 3D structures [27,28,29]. Melamine is named the formaldehyde-melamine-sodium bisulfite copolymer that can be shaped in fiber foam or sponge [30]. Melamine sponge (MS) is a commercial material with excellent properties such as low density, elasticity, and chemical stability. It is widely used, among else, as a heat insulator or sound absorber [31]. Almost exclusively in the literature, GO has been combined with MS with multistep processes that include the deposition of GO on MS, reduction of GO to the reduced graphene oxide (rGO), and stabilizing of the system with an extra polymer. MS doped with graphene derivatives combines ideally the superior elasticity and the porosity of the MS and the electrical conductivity of the graphene derivatives. The hybrid structures obtained are highly suitable for strain sensors with various applications, such as in flexible and wearable devices [4,5,6,32]. In addition, the porous structure of MS and the hydrophobic-lipophilic character of graphene derivatives are highly favoring the separation of organic liquids from water and could be used as absorptive material for the selective removal of organic liquid pollutants from water [32].

This article used a hydrophilic hybrid of pristine graphene nanosheets (G) with functionalized Multi-Walled Carbon Nanotubes (MWNT-*f*-OH) mixed with cellulose to dope MS. The product was a highly conductive and stable, piezoresistive composite that could be used as a susceptible flexible strain sensor. The hydrophilic G/MWNT-*f*-OH hybrid mixed with cellulose was deposited homogeneously between the melamine fibers of MS offering high conductivity. Cellulose was proved to be a necessary component to stabilize the graphene hybrid in the MS skeleton.

## 2. Materials and Methods

**Preparation of pristine graphene** [7]. A total of 100 mg of Graphite (Aldrich) was dispersed in 300 mL of DMF (Fisher Scientific) and sonicated for 6 h. The upper 270 mL of the suspension was isolated after overnight precipitation of the unexfoliated graphite. The graphene dispersion was concentrated with the flash evaporator.

**Synthesis of MWNT-*f*-OH** [7]. Pristine MWNTs (Aldrich; purity +95%, OD = 20–30 nm, length = 0.5–2 mm), 3,4-dihydroxybenzaldehyde (500 mg, Alfa Aesar) and N-methylglycine (500 mg, Alfa Aesar) were suspended in DMF (200 mL, Fisher Scientific). The mixture was heated at 120 °C for 5 days, and the solid part was separated by filtration through a PTFE membrane filter and washed with DMF and ethanol to remove organic byproducts. The purified product was redispersed in ethanol.

The G/MWNT-*f*-OH hybrid was prepared according to [7] by mixing a graphene dispersion in DMF (3.42 g L^−1^, 7 mL) and a MWNT-*f*-OH dispersion in ethanol (2.4 g L^−1^, 2.5 mL). The mass ratio G/MWNT-*f*-OH was 8:2. The mixture was stirred overnight, and then the hybrid was isolated by centrifugation (14,000 rpm, 10′). The hybrid G/MWNT-*f*-OH was then mixed with an aqueous solution of cellulose (15 g L^−1^, MW:250000, Sigma Aldrich) and drop-casted on a cubic piece of melamine sponge (10 mg) provided from the market. The modified sponge was then dried on a vial (2 h at 65–75 °C) and in a sealed bottle at 100 °C for 24 h. Three different samples were prepared according to the ratios in Table 1.

Scanning electron microscopy (SEM) was carried out on a Zeiss EVO-MA10 (Carl Zeiss Microscopy GmbH, Jena, Germany) operating at 12.05 kV. The samples for SEM microscopy were thin slices cut by a razor from the middle of the cubic MS pieces. For their mechanical and electrical characterization, the doped MS samples were placed between two horizontally oriented ITO glasses (2.5 cm × 7.5 cm). For the stability and recyclability measurements, the doped MS samples were compressed to a strain up to 50% and released for 12 cycles by applying a 2 N force vertically.

Compressive strain values were recorded manually by applying gradual pressure to the MS samples. The resistance of the MS samples was measured using the ITO glasses as electrodes after being compressed by a two-point probe system (Pro4 Resistivity System, Lucas Labs, Gilroy, CA, USA) and a Keithley 2400 Source Meter.

For the assessment of the sensor, two characteristics were also estimated. The gauge factor (GF) [4,33] and sensitivity (S) are defined by Equations (1) and (2) respectively.

GF = (ΔR/R_0_)/(ΔL/L_0_) (1)

S = (ΔR/R_0_)/ΔP (2)

where R and ΔR are the resistance and the change, L_0_ and ΔL are the lengths and the change after compression, respectively. ΔP is the change of the pressure. R was measured as described above, and L was measured manually. P was estimated by multiplying the applied force (2 N) with the surface area of the MS sample (1 cm^2^).

## 3. Results and Discussion

Hybrid G/MWNT-*f*-OH has been shown as a highly conductive and hydrophilic nanomaterial that can effectively be mixed with water-based polymers affording highly conductive composites [7,34]. Due to these favorable characteristics, G/MWNT-*f*-OH hybrid was selected here to dope MS samples, affording elastic and conductive polymer composites. The G/MWNT-*f*-OH hybrid was prepared using pristine graphene nanosheets (G) and chemically modified MWNTs (MWNT-*f*-OH) by a simple procedure as described elsewhere [7]. It was then mixed with cellulose in water, and the product was used to impregnate cubic pieces of MS samples with three different mass ratios regarding MS and the hybrid G/MWNT-*f*-OH.

**Figure 1:**
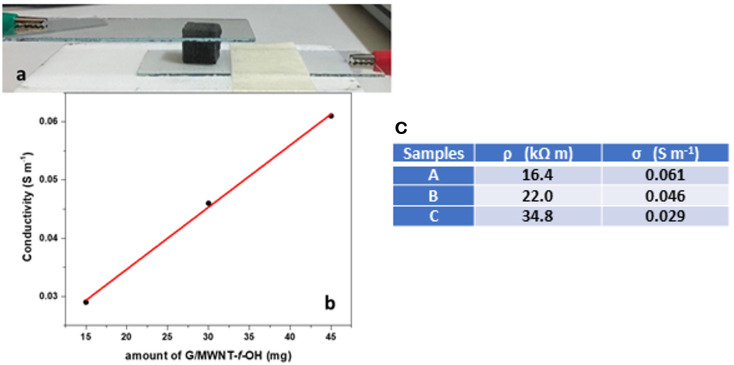
(**a**) A piece of MS impregnated with the composite hybrid G/MWNT-*f*-OH mixed with cellulose between ITO covered glass slides; (**b**) the conductivity of the MS samples vs. the amount of the conductive component, G/MWNT-*f*-OH; (**c**) the measured values of resistivity and conductivity of samples A–C.

The conductivity of the doped MS composites ranged between 0.029 and 0.061 S m^−1^, linearly analogue to the amount of the G/MWNT-*f*-OH hybrid, which was the conductive component (see Figure 1b,c). The measurements were performed using pieces of doped MS with dimensions of 1 cm × 1 cm^2^ between two horizontally oriented ITO glasses as electrodes (see Figure 1a). The linear relationship between the mass of the conductive component G/MWNT-*f*-OH and the conductivity indicated the homogenous dispersion of the hybrid G/MWNT-*f*-OH in the internal of the MS. The pure MS sample showed no detectable conductivity and is reasonably considered an insulator. Thus, the conductivity of the doped MS is attributed to electrical pathways that formed during the incorporation of the G/MWNT-*f*-OH hybrid between the fibers of MS.

**Figure 2:**
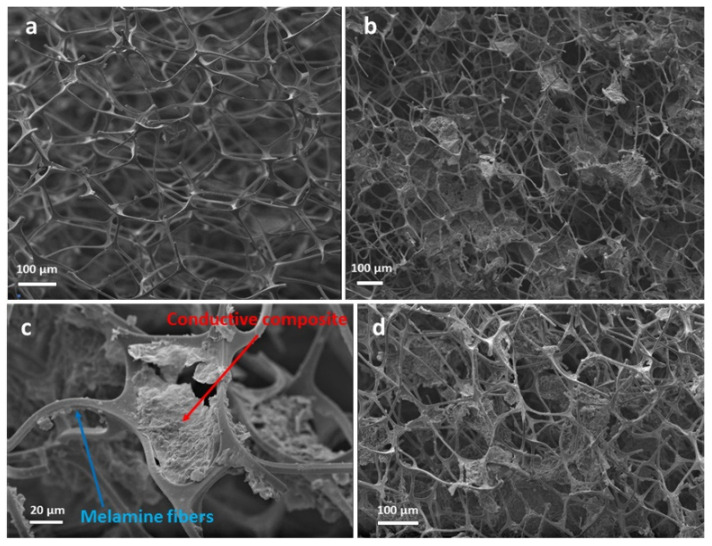
SEM images of the tomographies of (**a**) pure MS sample; (**b**–**d**) MS sample impregnated with the G/MWNT-*f*-OH/cellulose nanocomposite.

The microscopic analysis of the graphene-hybrid-doped MS samples was based on SEM images of the tomographies of the samples. SEM analysis revealed that the wide network of melamine fibers of the pure MS (Figure 2a) was partly covered by the G/MWNT-*f*-OH/cellulose nanocomposite. As shown in Figure 2b–d, the composite of hybrid G/MWNT-*f*-OH with cellulose was rather successfully entrapped between the melamine fibers than wrapped around them.

**Figure 3:**
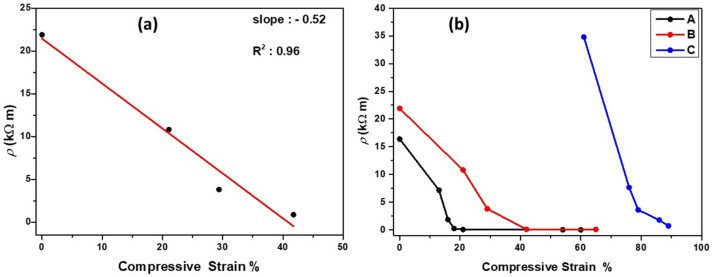
(**a**) Linear relationship between electrical resistivity and compressive strain of sample B. (**b**) The electrical resistivity of the doped MS samples versus compressive strain.

The G/MWNT-*f*-OH-doped MSs combine the elastic character of the melamine fiber network with the high conductivity of the G/MWNT-*f*-OH hybrid. The role of cellulose here was to function as a binder between the G/MWNT-*f*-OH hybrid and the melamine fibers. Impregnation of the MS directly with the G/MWNT-*f*-OH hybrid led to unstable structures where the carbon material was easily removed after a few compression cycles. As shown in Figure 3b, the electrical resistivity (*ρ*) of the three G/MWNT-*f*-OH-doped MS samples decreased remarkably by compressing. The compression here increased the interconnections between the graphene/CNTs nanostructures multiplying the number of the electrical pathways. The relationship between *ρ* and the compressive strain showed linearity at different values of the compression strain between the three MS samples. Τherefore, G/MWNT-*f*-OH-doped MS samples could function as effective piezoresistive sensors. For the most conductive-doped MS sample (**A**), *ρ* decreased linearly between 0 and 20% of the strain, while for the less conductive-doped MS sample (**C**), the linear area was between 60 and 80% of the stain. Instead, sample (**B**) showed the optimum broader linear area that ranged between 0 and 42%. The broad linear area between *ρ* and compressive strain indicated that the doped MS sample (**B**) was the most promising to work as a piezoresistive sensor.

**Figure 4:**
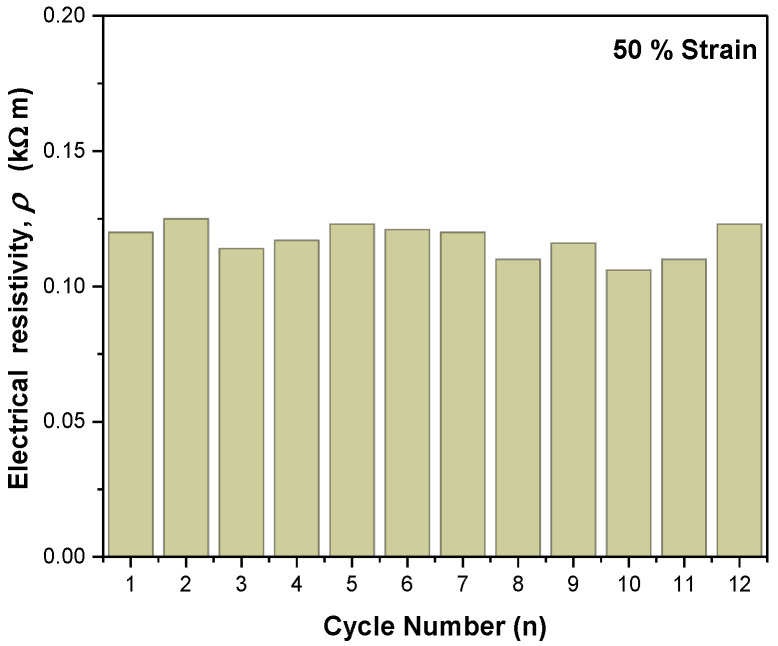
Stability of the electrical resistivity of sample MS **B**, at a compressive strain of 50%.

The G/MWNT-*f*-OH-doped MS samples were then compressed to a strain up to 50% and released for 12 cycles. The cycling stability of the most promising sample **B** was evaluated by measuring the electrical resistance of the compressed at 50% sample as a function of the number of cycles, as presented in Figure 4. The electrical resistivity of sample **B** compressed at 50% showed remarkable stability with a mean value of 117 Ω m and a standard deviation of 6 Ω m.

The promising electromechanical behavior of sample **B** was highly favorable for its use as a piezoresistive sensor. Piezoresistivity is a common sensing mechanism where the electrical resistivity of a structure changes during a mechanical strain of the structure [4,33,35]. The assessment of these types of sensors is based on the following criteria: the gauge factor (GF) or the sensitivity (S) as defined by Equations (1) and (2) (see Materials and Methods) and the applied pressure range. The gauge factor (GF) and sensitivity (S) of sample **B** were estimated to be 1.99 and 0.050 kPa^−1,^ respectively, while the very wide detection area ranges from 0 to 50 kPa [27,36].

## 4. Conclusions

An all-carbon hybrid made by pristine graphene nanosheets and organically modified MWNTs were finely incorporated into melamine sponges using water dispersion. Cellulose was also used to stabilize the deposition of the hybrid nanostructures between the melamine fibers. The prepared graphene-doped MS composite was highly flexible and stable with high conductivity and piezoresistive behavior. Based on the recent literature [4,36], it is concluded that a hydrophilic graphene hybrid can be used alternatively to GO to dope MS affording composite with great potential as a strain sensor since it combines a wide detection range with high sensitivity.

## Figures and Tables

**Table 1:** The ratio of the as-prepared modified melamine sponges.

Samples	Melamine Sponge (mg)	G/MWNT-*f*-OH (mg)	Cellulose (mg)
A	10	45	10
B	10	28	10
C	10	15	10

## Data Availability

Not applicable.
